# Polytraumatization in young male refugees from the Middle East and its association with internalizing and externalizing symptoms

**DOI:** 10.1186/s13034-021-00428-9

**Published:** 2021-12-17

**Authors:** Usama EL-Awad, Tilman Reinelt, Johanna Braig, Hannah Nilles, Denise Kerkhoff, Pia Schmees, Jana-Elisa Rueth, Atefeh Fathi, Mira Vasileva, Franz Petermann, Heike Eschenbeck, Arnold Lohaus

**Affiliations:** 1 Faculty of Psychology and Sports Sciences, University of Bielefeld, P.O. Box 10 01 31, 33501 Bielefeld, Germany; 2 Center for Clinical Psychology and Rehabilitation, University of Bremen, Grazer Str. 6, 28359 Bremen, Germany; 3 University of Zurich and University Hospital Zurich, Zurich, Switzerland; 4 Department of Educational Psychology and Health Psychology, University of Education Schwäbisch Gmünd, Oberbettringer Str. 200, 73525 Schwäbisch Gmünd, Germany; 5 Center for Psychosomatic Psychotherapeutic Rehabilitation, Luisenklinik, Paulinenstraße 21, 70178 Stuttgart, Germany; 6 Child and Community Wellbeing Unit, Melbourne School of Population and Global Health, University of Melbourne, 207 Bouverie St, Carlton, VIC 3053 Australia

**Keywords:** Refugee adolescents, Polytraumatization, Internalizing symptoms, Depression, Anxiety, Externalizing symptoms, Potentially traumatic events

## Abstract

**Background:**

Young Middle Eastern male refugees are currently among the most vulnerable groups in Europe. Most of them have experienced potentially traumatic events (PTEs) such as rape, torture, or violent assaults. Compared to their peers, young refugees suffer more from internalizing and externalizing symptoms, especially when unaccompanied. Little is known about the cumulative impact of experiencing different types of PTEs on mental health outcomes (polytraumatization) of young male refugees from the Middle East. We investigated (1) whether there is a dose–response relationship between multiple PTE types experienced and mental health outcomes, (2) whether individual types of PTEs are particularly important, and (3) to what extent these are differentially associated with mental health outcomes among unaccompanied or accompanied peers.

**Methods:**

In total, 151 young Middle Eastern male refugees in Germany (*M*_age_ = 16.81 years, *SD*_age_ = 2.01) answered questionnaires on PTEs, mental health, and post-migration stress.

**Results:**

Hierarchical regression analyses revealed, while controlling for age, duration of stay, unaccompanied status, and post-migration stress, (1) a dose–effect between PTE types on both internalizing and externalizing symptoms. Moreover, (2) regarding internalizing symptoms, violent family separation and experiencing life-threatening medical problems were particularly crucial. The latter was driven by unaccompanied refugees, who also reported higher levels of substance use.

**Conclusions:**

The results extend findings from the literature and suggest that not only may greater polytraumatization be related to greater depression among refugees, but also to a range of other mental health outcomes from the internalizing and externalizing symptom domains. Furthermore, the results highlight the mental health risks that unaccompanied and accompanied refugee adolescents face after exposure to PTEs, and provide information for practitioners as well as researchers about event types that may be particularly relevant.

**Supplementary Information:**

The online version contains supplementary material available at 10.1186/s13034-021-00428-9.

## Background

By mid-2020, the number of refugees worldwide was over 80 million, of whom about 40% lived outside their home countries [59]. In the record year of 2015, with around 890,000 people, more refugees arrived in Germany than ever before [5]. Adolescents and young adults formed the largest group and nearly 80% of them were male [5]. Most refugees came from Middle Eastern countries such as Syria and Afghanistan. Complex conflicts such as the Syrian civil war or violent unrest in Iraq or Afghanistan contributed to making home a life-threatening place. Before and during their flight to Europe, most young refugees were exposed to potentially traumatic events (PTE) such as torture, rape, and other aversive situations associated with violence [7]. These events have occurred, for example, on the Mediterranean route, which is usually boarded in substandard boats, or on the Balkan route [51]. Some of the young refugees experienced violence at the hands of smuggling gangs or by confrontation with border police [28, 58]. In mainland Europe, overcrowded temporary refugee camps with poor living conditions contributed to an increased risk of experiencing further traumatic events [31]. In 2017, for example, 13% of adolescents and children in Greek refugee camps were found to be malnourished, leading to stunting in some cases [26]. Young refugees are thus among the most vulnerable groups in any case, especially if they are unaccompanied. According to studies, up to 97% have had traumatic experiences, having been exposed to dangers almost without protection [63].

## Mental health of young Middle Eastern refugees in Europe

For children and adolescents, experiencing PTEs in the post-migration period can severely disrupt the developmental process into adulthood and lead to long-lasting deterioration in mental health outcomes [23]. In the host country, the prevalence of post-traumatic stress disorder (PTSD) varies from 40% to 60% depending on the study country, study design, and type of measurement [32, 62]. Although study findings are inconsistent, it is recognized that trauma-related disorders such as PTSD are prevalent among young refugees [29]. In particular, unaccompanied refugee youth suffer more from internalizing symptoms such as depression and anxiety compared to their non-refugee peers [7, 41]. In this regard, unaccompanied male refugees are considered a high-risk group who are more traumatized and exhibit more post-traumatic stress, increased stress perception, and poorer internalizing symptoms compared to accompanied peers [19].

In addition, unaccompanied peers in particular have been found to have higher levels of externalizing symptoms [32]. Among young male refugees from the Middle East who have arrived in Europe since 2015, externalizing symptoms such as rule-breaking behavior may be directly related to war experiences [2].

## Polytraumatization and correlates of mental health

Regarding the long-term consequences of these experiences, research on refugees has indicated a robust association between the variety of PTEs (larger number of different event types) experienced and mental health outcomes (e.g., Catani et al. [13] and Schauer et al. [49]). This association follows a building block-like dose effect, that mental health deteriorates and PTSD symptoms occur with cumulative trauma exposure [49]. The greater the variety of experienced traumatic events, the worse the mental health outcomes [13]. This is known as polytraumatization, as study results showed that young people who experienced multiple types of potentially traumatic events were more likely to suffer from mental health problems such as depressive symptoms and PTSD symptoms than young people who frequently experienced traumatic events of a single type (e.g., Finkelhor et al. [23]).

However, little is known about the extent to which the different types of PTEs, individually or when multiple event types occur simultaneously, influence the later mental health outcomes of young refugees. Differences in refugee history and culture between groups for young individuals appears to complicate research on this topic [53]. Age effects pose another challenge to the question of the weight of each stressor, as adolescents may not necessarily show the effects of traumatic experiences immediately afterwards or may show them in different ways depending on their developmental stage. While younger traumatized adolescents up to age 12 are more likely to suffer from internalizing symptoms, traumatized adolescents 13 years and older are more likely to exhibit externalizing symptoms classified as oppositional defiant behavior or conduct disorder and substance use [22].

Examining the relationship between mental health outcomes and the cumulative as well as the individual effects of different PTE types could provide valuable information for practitioners to focus attention on treatment priorities. In addition, it is likely that there are interactions between different types of events [30]. In this regard, it can be hypothesized that refugee adolescents who have experienced war and armed conflict are more likely to be victims of cumulative types of PTEs (e.g., war-related traumatic events). For example, it is conceivable that events leading to the forcible separation of young refugees from their families may result in a lack of protection and social support. This can potentially increase the risk of experiencing further PTEs and, at the same time, exacerbate the negative mental health effects of these experiences [17]. In addition to severe primary trauma and loss-related disorders (e.g., traumatic childhood grief, see Mannarino and Cohen [36]), loss of family support could indirectly lead to long-term disturbances in autonomy or identity development [9, 37], most notably reflected in increased rates of depression [16].

## Post-migration stress and mental health in young Middle Eastern refugees

Recent studies showed that the state of mental health among young refugees also depends on the level of stress in the host country [14]. In fact, post-migration stress, i.e., the tensions and strains caused by conflicts in adapting to the new country or by contact with the new culture and society, could explain up to 43% of the variance in internalizing symptoms [34]. For example, discrimination can be a major source of stress for Middle Eastern refugee youth, who sometimes find themselves in parts of societies that have harbored prejudice against young Muslim men since September 11 [38]. Hostile attitudes can make them feel unwelcome in the new environment [56]. In addition to pre-existing mental health problems due to stressful experiences prior to migration, rejection by the host society may cause young refugees to experience more severe depressive symptoms [20]. Furthermore, communication difficulties due to lack of knowledge of the host country's language can contribute to social isolation, especially in the first years after arrival [8]. On the other hand, it is also possible that psychological impairments due to pre-migration stress may prevent or impede learning the language of the host society [33]. At the same time, this makes it more difficult for adolescents to connect with local peers. In particular, unaccompanied adolescents are unlikely to be able to fully communicate their needs to their caregivers due to the language barrier [44]. In this context, separation from family may be associated with greater post-migration stress for unaccompanied young refugees than for accompanied peers [52]. In addition, the everyday life of young refugees is initially accompanied by a stressful asylum procedure in which fears about the future may arise [35]. In view of the asylum status and the associated uncertainty as to whether there is a right to stay, greatly increased anxiety symptoms can occur [43]. Poverty due to a poor financial situation can lead to frustration, especially during the processing of the asylum application, as there are no easily accessible work permits for refugees in European countries during this time [21].

Focusing exclusively on the relationship between experienced PTEs and mental health outcomes without considering current sources of stress would therefore fall short and yield biased results [47]. At the same time, it should be noted that some post-migration stressors disappear or have a lesser impact over time, e.g., reduced communication difficulties due to improved language skills in the host country language or reduced problems with the asylum process after being granted the right of residence. In this context, the length of stay in the host country seems to play a role in adolescent refugees’ perception of stress [24].

## Aims of this study

The present study aims to investigate the association between potentially traumatic events and mental health outcomes in male refugees from the Middle East who arrived in Germany during the refugee influx between 2015 and 2020. Since female refugee youths were hardly represented in Germany at the time of the first data collection in 2017, this article focuses only on unaccompanied and accompanied male adolescents and was guided by three main research questions: (1) Is there a dose–response relationship between the number of different potentially traumatic event types experienced (polytraumatization) and mental health outcomes (internalizing and externalizing problems) among young refugees, taking into account post-migration stress, age effects, length of stay, and unaccompanied status? (2) Do individual types of potentially traumatic events stand out in their association with young refugee mental health outcomes when a range of stressor types are considered simultaneously? (3) Are there specific potentially traumatic events that are differentially associated with mental health outcomes for unaccompanied versus accompanied refugees?

## Method

### Participants

The data was collected in Germany as part of two projects that examined the mental health of young refugees between 2017 and 2020, namely (1) BREfugee and (2) YOURHEALTH (subproject YOURGROWTH). The focus in the BREfugee project was on the largest refugee group in Germany (in addition to Middle Eastern migrant youth and native peers) at the time of data collection between 2017 and 2019, which consisted of male refugee adolescents from the Middle East (Syria, Iraq, Afghanistan, Iran, and Palestinian origin; *N*_BREfugee_ = 75). In YOURGROWTH, the focus was on the longitudinal survey of underage and adolescent Middle Eastern refugees from Syria, Iraq and Afghanistan at the first time of measurement in 2019 and 2020 (*N*_YOURGROWTH_ = 270 were available at the time of writing). To be able to reliably ensure correct assignment to the refugee group, all participants in both projects were asked for what reason they had left their home country. Participants who did not indicate a reason for flight as well as flight status were excluded from the study, as were participants from non-war countries or other than the specified Middle Eastern countries. The exclusion of participants from non-war countries achieved the composition of a reasonably homogeneous group in terms of pre-migration experiences. Thus, confounding with regard to potentially traumatic experiences can be excluded to a greater extent. Merging the data of both projects yielded a total sample size of *N* = 233, while after excluding cases due to missing values (see “Statistical analysis”), *N* = 151 participants aged between 14 and 22 years (*M*_age_ = 16.81 years, *SD*_age_ = 2.01 years) remained in the final sample. There were no significant age differences (only comparable variable) between participants who were excluded from the study and those who remained in the study, *t*(231) = 0.17, *p* = 0.864. The mean number of PTEs experienced as well as the internalizing and externalizing symptomatology did not differ significantly between the two project data sets, and a zero intraclass correlation coefficient suggested that there was no clustering underlying the projects in the merged data. On average, the participants had been in Germany for approximately 3 years and 1 month (*SD*_stay_ = 1.24 years). Additional file 1: Table S1 shows socio-demographic data of the participants.

### Procedure

After the participants, their parents and/or guardians had signed the written consent form, participants were tested either in a German research center or in the premises of migration associations (Project 1) or in school buildings or refugee accommodations (Project 2). In both projects, data collection was anonymous, and participation was voluntary. To gain the trust of the participants, it was explicitly pointed out that the study was conducted for purely scientific purposes. The participants filled out questionnaires individually or in a group setting on a computer or with tablets. Each complete session took up to three hours in total, while the part relevant to this study took about 25 min. In the preparatory phases of the projects, all measuring instruments were translated and back-translated [11] into the languages of the refugee group (Arabic, Farsi, Dari, Kurmanji, Pashto, and Sorani). In addition, in most of the sessions an accompanying native speaker was present and responded to questions of the participants in their first language. In the case of illiterates present among the participants, the interpreter assisted the young person in understanding and answering the questions. However, this was only the case for one participant. All procedures in both projects were approved by the local ethics committees of the universities of Bremen and Bielefeld before the data-collection started.

### Measures

#### Types of potentially traumatic events

The Stressful Life Events Screening Questionnaire (SLESQ; [25]) is a self-report screening tool that quantifies types of experienced potentially traumatic events. The questionnaire records events that have happened to the participant by providing a list. This list includes events from the areas of family-related traumatic events, traumatic bereavement, life-threatening illness or medical trauma, accident and natural disaster experiences, violent and sexual assault, and other traumatic experiences. It comprises a total of 13 items, 10 of which are assigned to individual experienced event types, such as traumatic bereavement and violent assaults (PTE 1 to PTE 10 in Table 1), two to general types of traumatic events (PTE 11 and PTE 12 in Table 1), and one consisting of an open-ended question about unlisted experienced events. For the purposes of this study, the open-ended question was excluded from the analysis. Depending on the respective analysis, the other items (*yes*/*no* response scale) were either used as single items or added together to form a total sum score of the experienced events. Goodman et al. [25] state good test–retest reliability (median kappa = 0.73) and adequate convergent validity with a detailed interview (median kappa = 0.64).

**Table 1:** Various types of potentially traumatic events derived from the SLESQ scale

Item	Category/type of event	*N* = 151 *n* (*%*)
*Category 1*	*Family-related traumatic events*	83 (55.0)
PTE 1	Change in the family situation in the last year	76 (50.3)
PTE 2	Separation from the family against the will of the young refugee, for example by the police, soldiers or strangers	28 (18.5)
*Category 2*	*Traumatic bereavement*	77 (51.0)
PTE 3	Death of a loved one	77 (51.0)
*Category 3*	*Life-threatening illness/medical trauma*	31 (20.5)
PTE 4	Experiencing a life-threatening medical problem	31 (20.5)
*Category 4*	*Experience of accidents*	54 (35.8)
PTE 5	Involvement in a serious accident	54 (35.8)
*Category 5*	*Experience with natural disasters*	41 (27.2)
PTE 6	Experiencing a natural disaster	41 (27.2)
*Category 6*	*Violent assaults*	110 (72.8)
PTE 7	Experiencing war or armed conflict	101 (66.9)
PTE 8	Experiencing gunshots, punches, kicks or other torments	56 (37.1)
*Category 7*	*Witnessing violent assaults*	65 (43.0)
PTE 9	Witnessing other people being tormented	65 (43.0)
*Category 8*	*Sexual assaults*	16 (10.6)
PTE 10	Being a victim of sexual assault or rape	16 (10.6)
*Category 9*	*Other traumatic experiences*	86 (57.0)
PTE 11	Experiencing other things that are perceived as dangerous	70 (46.4)
PTE 12	Experiencing other things that are perceived as dangerous to others	69 (45.7)

*PTE* potentially traumatic event (derived from SLESQ)

#### Mental health

The Hopkins Symptom Checklist-37A (HSCL-37A; [6]) showed good construct, content and criterion validity in previous studies, and contains 37 items (α = 0.95) in five subscales: depression (15 items, α = 0.91), anxiety (10 items, α = 0.87), conduct disorder (5 items, α = 0.75), substance use (5 items, α = 0.78), and oppositional defiant disorder (2 items, α = 0.55). Furthermore, the subscales depression and anxiety can be combined into “internalizing symptoms” (α = 0.94), while the remaining subscales make up the subscale “externalizing symptoms” (α = 0.85). All Cronbach’s alpha values reported above are from the present study and are in line with the alpha values reported for the original questionnaire (cf. [6]). Participants rated the items on a 4-point scale (*never* to *always*), based on the frequency of the symptoms experienced.

#### Post-migration stress

In both projects, participants indicated the extent of perceived migration-related stress from various sources across the last 12 months on a 5-point Likert scale. In the first project, the items were taken from the revised Post Migration Living Difficulties (PMLD; [54]) and in the second project from different post-migration scales, including the PMLD. Five matching items included in both projects (α = 0.68) were summed up and used as a covariate in this study, including the following domains: (1) language and communication difficulties, (2) processing the asylum application, (3) uncertainty and fear about residence permit, (4) isolation and exclusion, and (5) financial difficulties. For this item set, measurement invariance (MI) across both projects was tested using multi-group confirmatory factor analysis (CFA). Full metric MI was found, which allows using the items across the projects, configural invariance: comparative fit index (*CFI*) = 0.90, root mean square error of approximation (*RMSEA* = 0.072; changes of fit measures on metric invariance level: Δ*CFI* = − 0.002, Δ*RMSEA* = − 0.015.

### Statistical analysis

Data were analyzed using R 4.0.4 [46] and IBM SPSS 27, assuming a significance level of 5%. Missing values (2.7% of the data) were dealt with using the R package MICE 3.13.0 [60], with 85% of the cases being due to missing values in the SLESQ responses. Blockwise multiple imputation was used to substitute missing data with the corresponding estimation methods (multiple logistic regression imputation for binary data and predictive mean matching for ordinal data). Variables representing the subscales’ sum scores were formed using passive multiple imputations (see van Buuren and Groothuis-Oudshoorn [60]). Pearson product-moment correlations and point biserial correlations were calculated to examine relationships between the model variables. Multiple hierarchical regression analysis was used to examine the effects of cumulative types of PTEs (*Research Question 1*) and individual types of PTEs (*Research Question 2*) on mental health outcomes, taking into account age, length of stay, post-migration stress and unaccompanied status as covariates. The covariates were considered in the first step, with the model variables being included in further steps in order to investigate the increase in variance explanation. The analyses regarding Research Question 2 were preceded by a stepwise regression analysis based upon statistical significance to determine the best predicting subset concerning individual types of PTEs (PTE_i_) for internalizing and externalizing symptoms. The resulting subset of significant types of PTEs was then considered in the hierarchical regression analyses. In this way, potential confounding or suppression effects as well as multicollinearity problems can be avoided.

Finally, the extent to which the association between significant types of PTEs and mental health outcomes differs between unaccompanied and accompanied refugee youth was investigated by testing interactions in the final step of the hierarchical regression analysis, containing the unaccompanied status (binary coded with 0 = accompanied refugees, 1 = unaccompanied refugees), with PTE_i_, and PTE_i_ × unaccompanied status as predictors. An a priori power analysis yielded a power of at least 84% and higher for the given number of predictors and sample size regarding an expected medium effect size (*R*^2^ = 0.13) and type-I error probability of α = 0.05. For all regression analyses in the present study, 95% confidence intervals for coefficients were bias-corrected and accelerated (*BCa*) bootstrapped (based on 5000 resamples) to produce more accurate and robust estimators. The homogeneity of the error term variances was investigated with the modified Breusch–Pagan test and no heteroscedasticity issues could be determined regarding the analyses in the present study.

## Results

An overview of the types and corresponding categories of potentially traumatic events experienced by the refugee adolescents from the study sample is provided in Table 1. The vast majority has experienced war or armed conflict. More than half of youth have lost a loved one (PTE 3) or faced changes in the family situation in the year prior to the data collection (PTE 1). On average, the refugee youth experienced a total of 4 to 5 different events.

Looking at the aggregated number of various PTEs, just under 11% (*n* = 16) experienced events of one type. Approximately another 9% (*n* = 14) experienced two types of PTEs, while about 10% (*n* = 15) reported three types of PTEs. Approximately 15% of youth experienced four event types. Nearly half, and thus the majority, of all youth who fled (*n* = 75) reported having experienced five or more types of PTEs.

### Research Question 1: dose–effect relationship between variety of experienced potentially traumatic events and mental health outcomes among young refugees

Overall, there were positive correlations between the total PTE score and both internalizing, *r* = 0.36, *p* < 0.001, and externalizing symptoms, *r* = 0.25, *p* = 0.002 (see Additional file 1: Table S2 for further details). On closer inspection, the significant relationships were confirmed with all mental health outcomes, depression (*r* = 0.36, *p* < 0.001), anxiety (*r* = 0.31, *p* < 0.001) in the internalizing symptom area, and conduct disorder (*r* = 0.19, *p* = 0.021), substance use (*r* = 0.32, *p* < 0.001), and oppositional defiant disorder (*r* = 0.20, *p* = 0.014) in the externalizing symptom area.

The hierarchical regression analysis related to mental health outcomes regressed on the total PTE score while including age, length of stay, post-migration stress and unaccompanied status as covariates, resulted in consistently significant values (see Table 2), both for internalizing symptoms (Model 1) and for externalizing symptoms (Model 2). In both models, it was shown that the total PTE score significantly explained additional variance in mental health outcomes. Consistently, in the context of internalizing symptoms, PTE_total_ was a significant predictor of both depression (β_PTE total_ = 0.30, 95% *CI* [0.46, 1.40], *p* < 0.001 with Δ*R*^2^_depression_ = 0.09, Δ*F*(1, 143) = 17.80, *p* < 0.001 for the final step) and anxiety (β_PTE total_ = 0.27, 95% *CI* [0.20, 0.84], *p* = 0.002 with Δ*R*^2^_anxiety_ = 0.07, Δ*F*(1, 143) = 13.80, *p* < 0.001 for the final step). For the results on externalizing symptoms, PTE_total_ was a significant predictor for oppositional defiant disorder (β_PTE total_ = 0.20, 95% *CI* [0.02, 0.18], *p* = 0.011 with Δ*R*^2^_oppositional_ = 0.04, Δ*F*(1, 143) = 6.18, *p* = 0.014 for the final step), conduct disorder (β_PTE total_ = 0.17, 95% *CI* [0.01, 0.27], *p* = 0.039 with Δ*R*^2^_conduct disorder_ = 0.03, Δ*F*(1, 143) = 4.35, *p* = 0.039 for the final step), and substance use (β_PTE total_ = 0.24, 95% *CI* [0.07, 0.39], *p* = 0.003 with Δ*R*^2^_substance use_ = 0.05, Δ*F*(1, 143) = 11.11, *p* = 0.001 for the final step).

**Table 2:** Hierarchical regression analysis for the total number of potentially traumatic event types (derived from SLESQ) predicting mental health outcomes (HSCL-37A)

Variable	*B*	*SE B*	β	*BCa* 95% *CI*^a^	*p*	*Adj. R*^2^	Δ*R*^2^
Model 1: internalizing symptoms							
Step 1						0.22	
Age	− 0.27	0.52	− 0.04	[− 1.28, 0.76]	0.611		
Length of stay	− 0.75	0.75	− 0.07	[− 2.36, 1.21]	0.355		
Post-migration Stress	1.11	0.31	0.39	[0.55, 1.71]	< 0.001		
Unaccompanied status	6.01	2.71	0.21	[0.76, 11.18]	0.030		
Step 2						0.31	0.09
Age	− 0.47	0.49	− 0.07	[− 1.41, 0.51]	0.339		
Length of stay	− 1.24	0.72	− 0.12	[− 2.63, 0.37]	0.083		
Post-migration stress	1.07	0.32	0.37	[0.52, 1.17]	< 0.001		
Unaccompanied status	5.23	2.58	0.19	[0.21, 10.15]	0.043		
PTE total	1.48	0.36	0.30	[0.73, 2.16]	< 0.001		
Model 2: externalizing symptoms							
Step 1						0.13	
Age	− 0.22	0.18	− 0.09	[− 0.62, 0.14]	0.251		
Length of stay	− 0.20	0.32	− 0.05	[− 0.87, 0.59]	0.543		
Post-migration stress	0.37	0.16	0.33	[0.11, 0.65]	0.019		
Unaccompanied status	1.81	1.17	0.17	[− 0.65, 4.18]	0.137		
Step 2						0.17	0.04
Age	− 0.27	0.18	− 0.11	[− 0.65, 0.08]	0.148		
Length of stay	− 0.33	0.29	− 0.08	[− 0.94, 0.37]	0.275		
Post-migration stress	0.36	0.17	0.32	[0.09, 0.64]	0.034		
Unaccompanied status	1.61	1.13	0.15	[− 0.83, 4.04]	0.172		
PTE total	0.38	0.17	0.20	[0.01, 0.70]	0.034		

*N* = 148. **p < 0.01. ***p < 0.001. Model 1, Step 1: *F*(4, 144) = 11.58, *p* < 0.001. Model 1, Step 2: *F*(5, 143) = 14.11, *p* < 0.001; and Δ*F*(1, 143) = 18.58, *p* < 0.001. Model 2, Step 2: *F*(4, 144) = 6.67, *p* < 0.001. Model 2, Step 2: *F*(5, 143) = 6.91, *p* < 0.001; and Δ*F*(1, 143) = 6.80, *p* = 0.010. Model 1, *VIF* (maximum) = 1.32; Model 2, *VIF* (maximum) = 1.32

^a^Bias-corrected and accelerated bootstrap (*BCa*) confidence intervals (*CI*) based on 5000 resamples

**Fig. 1:**
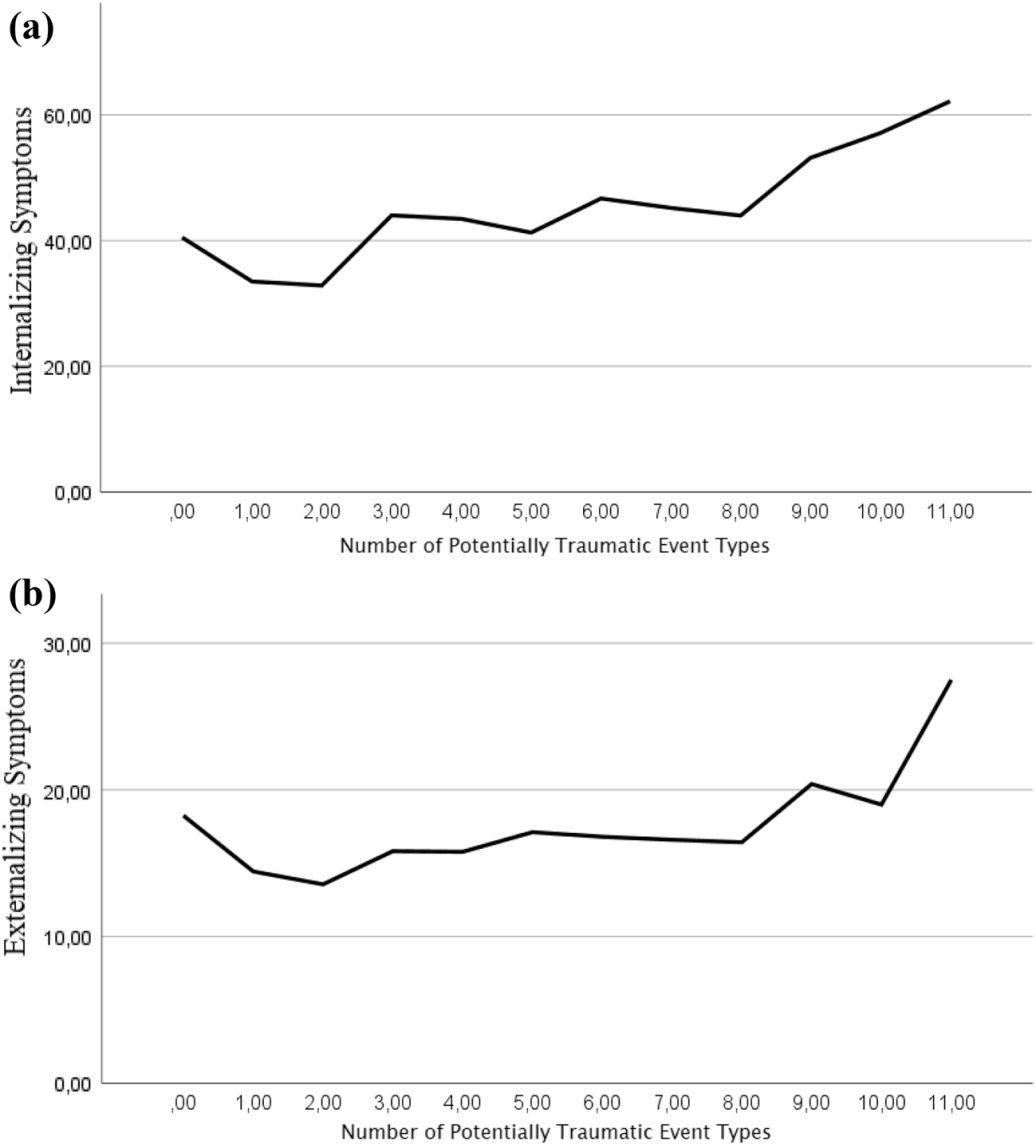
Graphical representation of the relationship between **a** internalizing and **b **externalizing mental health outcomes and the total number of experienced potentially traumatic event types among Middle Eastern male refugee adolescents in Germany

This indicates that the more different types of PTEs or corresponding categories are experienced, the greater the suffering from internalizing and externalizing symptoms. As Fig. 1 illustrates, the magnitude of internalizing and externalizing symptoms was associated with the number of different event types experienced, with a more distinct increase above a number of nine event types.

### Research Question 2: significant individual PTE types

The inter-item correlation matrix of the SLESQ-items and their relationship with the mental health outcomes is provided in Additional file 1: Table S3. A preceding stepwise regression analysis revealed for internalizing symptoms (adjusted *R*^2^ = 0.13, *F*(2, 148) = 12.03, *p* < 0.001) and externalizing symptoms (adjusted *R*^2^ = 0.08, *F*(2, 148) = 7.31, *p* = 0.001) that forcible separation from family (predicting internalizing symptoms: β_PTE 2_ = 0.31, *t*(148) = 4.00, *p* < 0.001; and externalizing symptoms: β_PTE 2_ = 0.23, *t*(148) = 2.91, *p* = 0.004) and life-threatening medical problems (predicting internalizing symptoms: β_PTE 4_ = 0.19, *t*(148) = 2.44, *p* = 0.016; and externalizing symptoms: β_PTE 4_ = 0.17, *t*(148) = 2.19, *p* = 0.030) were the only significant predictors.

Regarding *Research Question 2*, as can be seen from the results of the hierarchical regression analysis (Table 3; see Step 2 in Model 3 and Model 4), there were significant associations between this set of PTE types (resulting from the preceding stepwise regression analysis), that is violent family separation and living through a life-threatening medical problems, and mental health outcomes—independent of youth age, length of stay and post-migration stress. Repeating the regression analysis with the individual mental health outcomes in the internalizing symptom area yielded consistent results and revealed additional information. Experiencing family separation explained additional variance in depression (β_PTE 2_ = 0.18, 95% *CI* [0.34, 7.26], *p* = 0.030; adjusted *R*^2^_depression_ = 0.26, *F*(12, 135) = 4.55, *p* < 0.001) but not in anxiety. Instead, living through a life-threatening medical problem (PTE 4) seems to be a particularly strongly predictor of anxiety symptoms, β_PTE 4_ = 0.20, 95% CI [0.65, 4.55], *p* = 0.012 (adjusted *R*^2^_anxiety_ = 0.27, *F*(12, 135) = 4.63, *p* < 0.001).

**Table 3:** Hierarchical regression analysis for significant potentially traumatic event types (derived from SLESQ) predicting mental health outcomes (HSCL-37A)

Variable	*B*	*SE B*	β	*BCa* 95% *CI*^a^	*p*	*Adj. R*^2^	Δ*R*^2^
Model 3: internalizing symptoms							
Step 1						0.19	
Age	0.19	0.48	0.03	[− 0.73, 1.14]	0.695		
Length of stay	− 0.91	0.86	− 0.09	[− 2.52, 1.05]	0.281		
Post-migration stress	1.26	0.29	0.44	[0.71, 1.80]	< 0.001		
Step 2						0.25	0.07
Age	0.11	0.48	0.02	[− 0.90, 1.09]	0.834		
Length of stay	− 1.07	0.77	− 0.10	[− 2.54, 0.66]	0.166		
Post-migration stress	1.11	0.31	0.39	[0.55, 1.67]	< 0.001		
PTE 2 (family separation)	6.64	2.46	0.20	[1.56, 11.71]	0.009		
PTE 4 (medical problem)	5.00	2.32	0.16	[0.49, 9.59]	0.038		
Step 3						0.29	0.05
Age	− 0.05	0.49	− 0.01	[− 1.03, 0.92]	0.915		
Length of stay	− 0.70	0.78	− 0.07	[− 2.20, 1.05]	0.356		
Post-migration stress	0.96	0.33	0.34	[0.40, 1.56]	0.003		
Unaccompanied status	2.36	3.09	0.08	[− 4.10, 8.84]	0.447		
PTE 2 (family separation)	5.08	3.19	0.15	[− 1.20, 11.28]	0.110		
PTE 4 (medical problem)	1.50	2.56	0.05	[− 3.83, 6.78]	0.550		
PTE 2 × Unacc. Status	1.03	4.48	0.02	[− 8.46, 10.06]	0.812		
PTE 4 × Unacc. Status	11.62	4.18	0.22	[3.20, 19.19]	0.006		
Model 4: externalizing symptoms							
Step 1						0.12	
Age	− 0.08	0.20	− 0.03	[− 0.47, 0.33]	0.675		
Length of stay	− 0.25	0.32	− 0.06	[− 0.91, 0.51]	0.403		
Post-migration stress	0.41	0.15	0.37	[0.15, 0.72]	0.008		
Step 2						0.14	0.04
Age	− 0.10	0.20	− 0.04	[− 0.46, 0.29]	0.622		
Length of stay	− 0.29	0.30	− 0.07	[− 0.92, 0.40]	0.316		
Post-migration stress	0.37	0.16	0.34	[0.10, 0.70]	0.022		
PTE 2 (family separation)	1.75	1.17	0.14	[− 0.78, 3.98]	0.139		
PTE 4 (medical problem)	1.59	1.07	0.13	[− 0.24, 3.43]	0.139		
Step 3						0.19	0.07
Age	− 0.13	0.17	− 0.06	[− 0.46, 0.20]	0.448		
Length of stay	− 0.14	0.32	− 0.03	[− 0.78, 0.55]	0.660		
Post-migration stress	0.32	0.17	0.29	[0.05, 0.68]	0.049		
Unaccompanied status	0.74	1.28	0.07	[− 1.94, 3.05]	0.567		
PTE 2 (family separation)	2.06	1.43	0.16	[− 0.95, 4.79]	0.162		
PTE 4 (medical problem)	− 0.11	0.96	− 0.01	[− 2.02, 1.67]	0.915		
PTE 2 × Unacc. Status	− 1.79	2.21	− 0.10	[− 6.05, 2.75]	0.420		
PTE 4 × Unacc. Status	5.92	2.43	0.29	[0.98, 10.77]	0.023		

*N* = 148. **p < 0.01. ***p < 0.001. Model 1, Step 1: *F*(3, 145) = 12.79, *p* < 0.001. Model 1, Step 2: *F*(5, 143) = 10.85, *p* < 0.001; and Δ*F*(2, 143) = 6.50, *p* = 0.002. Model 1, Step 3: *F*(8, 140) = 8.52, *p* < 0.001; and Δ*F*(3, 140) = 3.63, *p* = 0.015. Model 2, Step 1: *F*(3, 145) = 7.45, *p* < 0.001. Model 2 Step 2: *F*(5, 143) = 5.94, *p* < 0.001; and Δ*F*(2, 143) = 3.17, *p* = 0.045. Model 2, Step 3: *F*(8, 140) = 5.43, *p* < 0.001; and Δ*F*(3, 140) = 3.96, *p* = 0.010. Model 1, *VIF* (maximum) = 2.36; Model 2, *VIF* (maximum) = 2.36

^a^Bias-corrected and accelerated bootstrap (*BCa*) confidence intervals (*CI*) based on 5000 resamples

However, regarding externalizing symptoms, the regression analysis did not yield significant results in terms of mental health prediction by specific PTE types on its own. This means that there was no significant additional prediction of externalizing symptoms from the set of SLESQ items in the regression analysis. Upon closer examination, this finding can be confirmed for the subscales, as in addition to post-migration stress, only length of stay was a significant predictor for the outcome oppositional defiant disorder, β_stay_ = − 0.17, 95% *CI* [− 0.35, − 0.01], *p* = 0.031 with adjusted *R*^2^_oppositional_ = 0.11, *F*(12, 135) = 2.27, *p* = 0.007 for the final step, indicating that the oppositional behavior decreased with longer stay. For conduct disorder, the results also showed no additional significant predictive power of any of the PTEs and other variables, but in the overall analysis, younger age appeared to be associated with fewer symptoms, β_age_ = − 0.20, 95% *CI* [− 0.38, − 0.06], *p* = 0.023 with adjusted *R*^2^_conduct_ = 0.10, *F*(12, 135) = 2.08, *p* = 0.014 for the final step. With respect to substance use, post-migration stress showed the only predictive power (β_post-migration stress_ = 0.34, 95% *CI* [0.06, 0.27], *p* = 0.003) beyond the other predictors in the final step (adjusted *R*^2^_substance use_ = 0.23, *F*(12, 135) = 3.91, *p* < 0.001).

### Research Question 3: differences between unaccompanied and accompanied refugee adolescents in terms of significant PTE types

When examining group differences between unaccompanied and accompanied refugees, it appeared for both internalizing and externalizing symptoms that experiencing a life-threatening medical problem was associated with significantly stronger symptoms for unaccompanied than for accompanied refugees (Table 3; see Step 3 in Model 3 and Model 4). These associations held for increased depressive symptoms (*b*_PTE 4 × unaccompanied_ = 8.08, 95% *CI* [2.23, 13.41], *p* = 0.015 with adjusted *R*^2^_depression_ = 0.24, *F*(6, 142) = 8.89, *p* < 0.001) as well as increased symptoms of anxiety (*b*_PTE 4 × unaccompanied_ = 6.25, 95% *CI* [3.10, 9.57], *p* = 0.001 with adjusted *R*^2^_anxiety_ = 0.29, *F*(6, 142) = 10.93, *p* < 0.001) in the internalizing symptom area for the unaccompanied youth. In the area of externalizing symptoms, the association was exclusively triggered by increased substance use in unaccompanied adolescents but not by other outcomes, *b*_PTE 4 × unaccompanied_ = 4.54, 95% *CI* [2.38, 6.66], *p* = 0.001 with adjusted *R*^2^_substance use_ = 0.34, *F*(6, 142) = 15.32, *p* < 0.001. Overall, unaccompanied refugees experienced approximately five PTEs on average (5.03), while accompanied peers experienced events averaging just over four PTEs (4.26), which, however, results in a non-significant mean comparison, *t*(147) = − 1.60, *p* = 0.111.

## Discussion

The present study aimed to examine in more detail the relationship between types of experienced potentially traumatic events and mental health outcomes among young male Middle Eastern refugees resettled in Germany. In short, the results revealed a dose–effect for both internalizing and externalizing symptoms depending on the number of experienced PTE types. In addition, exposure to violent family separation and life-threatening medical problems were particularly significant in predicting internalizing symptoms, with the latter especially important for unaccompanied refugees.

Nearly half of the study’s participants had experiences that summed up to five or more types of PTEs and fell into four or more event categories. Although this indicates polytraumatization among young refugees from the Middle East, the total number of various events, averaging four to five, was still lower than in other studies that surveyed refugee youth from the same countries of origin who arrived in Europe during the same period (e.g. Sleijpen et al. [55]). Since the participants of this study were on average 1 to 2 years older, one explanation could be that memories were repressed with increasing age when the events occurred in young years and were therefore only partially reported due to retrospective measurement [48]. Another explanation may be found in the different measurement tools used in the studies to capture the types of PTEs in refugee youth. For example, differences are found particularly in terms of the lists of event types used, as these were defined more narrowly in categories such as traumatic bereavement (e.g., Sleijpen et al. [55]). A narrower or broader list should not change the results in this study, as the measurement results are considered relative to their relationship with other variables. The prerequisite for this is an appropriate list that is able to capture typical types of PTEs in young refugees without missing frequent and representative event types or categories. If this requirement is not met, the dose–effect relationship between the number of experienced PTE types and mental health outcomes is likely to be underestimated.

Nevertheless, a dose–effect was demonstrated in the present study, suggesting that adverse mental health outcomes were associated with a larger number of various types of PTEs or categories, while current post-migration stress, length of stay, and varying age were taken into account (*Research Question 1*). In particular, the association with mental health deterioration appeared to increase more sharply after nine types of PTEs and slightly for less than nine event types. This finding is consistent with other studies stating that a certain cumulative trauma exposure (“trauma load”) is necessary to trigger mental health problems concerning PTSD (e.g., Schauer et al. [49]). The present findings, however, imply that the experience of multiple types of PTEs has an additive effect on various mental health outcomes. While a study with young Syrian refugees in Turkish refugee camps could find a similar effect on depression [42], the results here suggest that this also applies to other internalizing and externalizing symptoms.

However, as the SLESQ is a conventional traumatic event type checklist, it should be kept in mind that the total number of PTEs is not quantified by counting the occurred traumatic events per se, retrospectively recalled by the adolescents. Nevertheless, it is likely that particularly in refugee pre-migration contexts, there are risks for (a) a higher number of potentially traumatic events as well as (b) a greater variety of these events, thus leading to a strong correlation between the count and variety [45]. Especially for adolescents who have survived war and other traumatic events, the variety score appeared to have strong predictive power with respect to mental health outcomes [13]. Moreover, the variety score might even be a stronger predictor when directly compared to the count, based on the possibility that a habituation effect could occur with certain—but not all—repetitive types of traumatic events (e.g., viewing corpses in war zones), attenuating the association with mental health [45]. Further, experiencing a higher variety of traumatic events can trigger a multitude of problems that need to be overcome simultaneously, which might lead to a range of deteriorated mental health outcomes [23]. For the refugee adolescents who participated in the present study, it was clearly shown that there is a reinforcing association between multiple types of traumatic experiences and poor mental health outcomes across all assessed symptom areas.

Nonetheless, analyzing significant individual types of PTEs in terms of mental health outcomes and their group-specific difference among unaccompanied and accompanied refugee adolescents (*Research Question 2*) provided a deeper look on the effects. The purpose of this examination was, on the one hand, to circumvent the problem found in the literature, where simply summing up all types of experienced PTE among refugee youth results in equal weighting of the event types [39]. On the other hand, it provided information on particular important event types that predict mental health outcomes in both unaccompanied and accompanied refugee peers. Accordingly, event types in the past surrounding the violent separation of adolescents from their families beyond other events were predictors for the emergence of depression in all refugee adolescents, whereas the experience of a life-threatening medical problem predicted anxiety symptoms particularly in unaccompanied peers.

Forcible separation from the family, even if temporary, puts unprotected young refugees at greater risk of experiencing further traumatic events with greater negative impacts on mental health [18]. Furthermore, deficits arise with the loss of socioemotional or economic support factors that normally come from the family and not only help to cope with experienced traumatic events, but also drive the normative development process of adolescents. Previous study results state that in the absence of adequate family support, victims are two to four times more likely to experience severe depression and anxiety after the traumatic event, because recovery is prevented, underscoring the buffering hypothesis of socioemotional support [61]. Developmental problems due to lack of contact with their family may in turn subsequently interfere with building relationships with peers and mentors and accepting socioemotional support, resulting in an increased risk for depression [52]. However, since the relationship between unaccompanied refugees and those participants who reported being forcibly separated from family is a moderate one (*r*_unaccompanied × PTE 2_ = 0.21, *p* = 0.011), it might be assumed that some of the participants experienced the separation only temporarily. This is strengthened by the fact that no significant group-specific interaction could be identified for family separation. It is possible that there might be a proportion of refugees who initially fled to Europe unaccompanied and whose families later moved to the host country before the data were collected. Furthermore, being unaccompanied does not directly mean that a violent separation from or loss of family has necessarily occurred—so while experienced events in PTE 2 (separation from family) are very likely for unaccompanied youth, they are not a requirement.

The finding of a striking association between life-threatening medical problems and internalizing symptoms among the young refugees can be attributed to unaccompanied adolescent refugees. Unaccompanied refugees showed more severe symptoms than their accompanied peers in both internalizing and, moreover, externalizing domains after experiencing medical events. It is reasonable to assume that the lack of family support experienced by unaccompanied refugees may impede their ability to cope with serious medical experiences through unavailable parental caring and assistance [15]. This may also explain the greater substance use in unaccompanied refugees compared with their accompanied peers, as the lack of family support or family control could lead to limitations in the youth's repertoire of adaptive coping strategies, which increases their vulnerability for using inappropriate coping strategies [12].

Nevertheless, it should be emphasized that the SLESQ does not provide information about the kind of life-threatening medical problems that are associated with increased mental problems in unaccompanied refugees. Thus, no precise statement can be made about how suddenly and intensively the medical problems occurred, whether hospitalization was necessary, or to what extent the medical problems were related to war experiences. While violent separation from family can be attributed to pre- and peri-flight circumstances, as such an experience is unusual for occurring in the host country, this cannot be clearly assumed for going through medical issues. It is, however, conceivable that life-threatening medical problems before and during flight are more likely to be due to war-related injuries. These occur suddenly and traumatize in a different and more unexpected way than sufferings from illnesses in the host country, where medical care may be more likely available and accessible. In addition, diseases in the host country can also be late effects of the war. Study findings demonstrate a higher rate of heart disease among Middle Eastern adolescent refugees due to war-related factors, such as malnutrition [3]. This could explain increased anxiety symptoms, as the significant association between heart disease and anxiety symptoms has been demonstrated at least in children and adolescents [40].

In summary, contextual factors providing information about the kind of medical problem and the persistence of anxiety symptoms as well as the place and time of the event could be relevant, as they contribute to the young refugee's perception. Thus, to shed more light on this finding, future longitudinal studies are needed that differentiate between diagnoses and generally ask for further relevant background information like time, place, and severity of PTEs. Moreover, differentiation of anxiety symptoms is likely to be essential to assess whether, for example, it is health anxiety, which is also suspected to be related to childhood trauma in non-refugees [57]. In the present study, health anxiety may possibly be inferred indirectly, albeit with uncertainty, because those participants who experienced a life-threatening medical problem simultaneously reported high levels of attacks of fright and panic (*r*_medical problem × panic_ = 0.38, *p* = 0.001) and fearfulness (*r*_medical problem × fear_ = 0.22, *p* = 0.006)—symptoms which are comorbid with health anxiety [1].

### Associations of pre- and post-migration stress with mental health

In addition, the negative association between post-migration stress and mental health outcomes is as least as important as the association of PTEs and mental health outcomes. However, it seems likely that the negative impact from the post-migration stress side decreases with the length of stay [24], but the trauma-related stress increases in the absence or incompleteness of appropriate treatments [48]. In particular, trauma-related stress might not show up until adulthood through increasing more severe health problems [22]. Thus, a conclusion that post-migration stress has at least as strong a negative association with mental health outcomes as traumatic experiences in the refugees’ childhood or adolescence would not only be premature, but a snapshot at the time of data collection. In addition, studies have postulated a relationship between potentially traumatic experiences and post-migration stress among refugee adolescents from the Middle East that were resettled in other European countries with cumulative effects on mental health [14]. Such a relationship could not be determined in the present study, suggesting that the relationships with mental health outcomes were rather independent of each other (*r*_PTE total × post-migration stress_ = 0.10, *p* = 0.241). However, this lack of evidence of a relationship between pre- and post-migration stress might be attributed to the distinct measurement procedures, namely that different pre- as well as post-migration stressors were recorded across the studies. On the other hand, the perception of post-migration stress depends on the refugee regulations (e.g., duration and prospect of the right to stay), laws (e.g., work permit issuance) and social views towards refugees (e.g., discrimination) of the respective country, so that the perception of stressors might already differ qualitatively between the samples.

## Strengths and limitations

The results of this study may not be generalizable to other resettlement communities if the characteristics of the pre-migration history (e.g., different flight routes, types of flight, duration of flight, etc.) and resettled youth (e.g., degree of stress due to flight history and coping options) differ. The mental health associated perception of experienced PTEs may differ in this sense, making mental health outcomes dependent on the group studied and its environment [50]. Nevertheless, because male adolescents from the Middle East made up a large portion of the refugee population in Germany and Europe at the time of the data collection, this study is representative in terms of the refugee population. The reason for the overrepresentation of male youths is presumably the dangerousness of the escape routes, which may discourage females to make the journey. However, the gender constellation among Middle Eastern refugee adolescents in European countries as Germany seems to have changed recently [4], so that the analyses from the present study should be extended to female participants, especially since the state of research regarding Middle Eastern female adolescents’ mental health is still very limited in European countries. An exception is provided by the study of Abu-Kaf et al. [2] who reported that female refugees from the Middle East in Greece experienced more community cohesion as well as stronger social support from aid organizations than their male peers. Nonetheless, study findings on traumatizing events in other cultural groups, for instance through exposure of missile attacks in southern Israel, suggest that female adolescents have stronger subsequent stress reactions than their male peers [10].

Furthermore, the cross-sectional nature of the study does not allow for causal conclusions but does provide insight into the complex interplay of pre-migration factors and contemporary mental health issues examined here. Longitudinal studies are needed to examine timely relationships, particularly the extent to which the relationship between pre-migration stress and mental health changes over time in the transition to adulthood.

In addition, this study uses screening instruments that do not allow for a definitive diagnosis, especially with regard to PTSD. However, the conceptualization of the study is aimed at examining a broader spectrum of symptoms with transdiagnostic markers. On the one hand, this is to take into account the high comorbidity of mental disorders, especially among refugees in adolescence, and on the other hand, to provide information for the development of prevention and interventions that achieve the improvement of several types of symptoms in equal measure.

Another limitation can be found in the multiple language versions of the scales used in the present analyses, in case participants might have understood the conceptualizations differently depending on their language. For the mental health and, to some extent, post-migration stress scales, results of measurement invariance tests between different cultural groups are available in another study—partly with the same groups and language versions used in the present analyses (see EL-Awad et al. [19]). These indicate that there were no significant differences in response behavior and language comprehension with respect to the questions. With respect to the SLESQ, no test for measurement invariance could be carried out due to the form of the scale as a checklist. It cannot necessarily be assumed that the events are correlated—especially not with regard to different groups. Nevertheless, the groups could differ by language context. However, studies using the SLESQ suggest that questions were predominantly understood similarly by participants in different language versions (e.g., Green et al. [27]).

Finally, it cannot be guaranteed that both projects from which the data used for the present analyses originate were comparable in all aspects regarding recruitment and data collection. Non-equivalent implementation could lead to bias in results. However, statistical tests do not provide any evidence that project-specific characteristics or differences exist with regard to the data.

## Conclusions and implications for future research

The results of the present study can inform practitioners and therapists where to place particular focus regarding mental health treatment for Middle Eastern male refugees in European countries and which adolescents are at particular risk based on their history of traumatic experiences. First of all, due to polytraumatization, special attention should be paid to refugee adolescents, especially those who are unaccompanied, but also those who have only been temporarily unaccompanied, e.g. by starting their flight alone. This study has shown that even the short-term separation from family can be associated with mental health problems among adolescent refugees. For this reason, the public strategy in dealing with adolescents separated from their families should always be to seek family reunification. For refugee adolescents who are in the host country with one or both parents, it can be assumed that care and support should accordingly be offered by holistically addressing internal family processes and conflicts, and post-traumatic stress disorders of the family members. Such programs must involve the family in solving the psychological problems of adolescent refugees. Beyond this, the results of this study suggest a particular risk to the mental health of youth who have experienced life-threatening medical problems. Although these medical problems could not be clearly categorized as a consequence of the war situation, there is evidence in the literature (e.g., Al-Ammouri and Ayoub [3]) that they could be. This should not be overlooked in mental health treatments and hence examined in more detail. In particular, with regard to substance use among unaccompanied refugee youth from the Middle East, this study provides evidence that, at least in the context of the participants, it was more likely to occur when a life-threatening medical problem was experienced. These associations need to be tested for causality in future large-scaled studies, in order to derive new treatment approaches for such resistant problems as substance use in unaccompanied refugee adolescents.

## Supplementary Information

**Additional file 1: Table S1.** Socio-demographic factors of participants. **Table S2.** Descriptive statistics and Pearson’s correlations for mental health outcomes (HSCL-37A) and PTE total (SLESQ). **Table S3.** Descriptive statistics and correlations for PTEs (SLESQ items), internalizing and externalizing symptoms (HSCL-37A subscale outcome).

## Data Availability

The data that support the findings of this study are confidential, but available from the corresponding author upon reasonable request.

## Abbreviations

PTSD: Post-traumatic stress disorder
PTE: Potentially traumatic event
CI: Confidence interval
CFI: Comparative fit index
RMSEA: Root mean square error of approximation
