# Supplementary material for: Polytraumatization in young male refugees from the Middle East and its association with internalizing and externalizing symptoms
Source: Child Adolesc Psychiatry Ment Health. 2021 Dec 17;15:75. doi: 10.1186/s13034-021-00428-9 (PMC8684214; doi:10.1186/s13034-021-00428-9)
Supplement: Supplementary file 1 — Additional file 1: Table S1. Socio-demographic factors of participants. Table S2. Descriptive statistics and Pearson’s correlations for mental health outcomes (HSCL-37A) and PTE total (SLESQ). Table S3. Descriptive statistics and correlations for PTEs (SLESQ items), internalizing and externalizing symptoms (HSCL-37A subscale outcome). [file 13034_2021_428_MOESM1_ESM.docx]

**Additional file 1**

**Polytraumatization in Young Male Refugees from the Middle East
and its Association with Internalizing and Externalizing Symptoms**

Child and Adolescent Psychiatry and Mental Health

[Author names edited out for blind review]

**################################################**

1. **Table S1**
2. **Table S2**
3. **Table S3**

**################################################**

Table S1

*Socio-demographic factors of participants*

| Factors | Refugee Adolescents |  |  |
| --- | --- | --- | --- |
|  | *N* = 151 |  |  |
| Age (in years), *M* (*SD*) | 16.81 (2.01) |  |  |
| Duration of Stay, *M* *(SD)* | 3.12 (1.25) |  |  |
| Unaccompanied (valid responses = 149), *n* (%) | 42 (28.2) |  |  |
| Country of origin (valid responses = 151), *n* (*%*): |  |  |  |
| Syria | 97 (64.2) |  |  |
| Afghanistan | 28 (18.5) |  |  |
| Iraq | 19 (12.6) |  |  |
| Others   (e.g. Palestinian descent) | 7 (4.7) |  |  |
| Education in host and origin country  in school years (valid responses = 149), *M* (*SD*) | 7.7 (2.9) |  |  |
| Formerly employment (valid responses = 137), *n* (*%*) | 76 (50.3) |  |  |
| Asylum status (valid responses = 116), *n* (*%*): |  |  |  |
| Seeking (not yet registered) | 32 (27.6) |  |  |
| Applied | 14 (12.1) |  |  |
| Granted | 67 (57.8) |  |  |
| Rejected  (tolerated stay) | 3 (2.5) |  |  |
| Religion (valid responses = 147), *n* (*%*): |  |  |  |
| Islam | 123 (83.7) |  |  |
| Christianity | 9 (6.1) |  |  |
| Atheism/  Agnosticism | 5 (3.4) |  |  |
| Others | 10 (6.8) |  |  |

Table S2

*Descriptive statistics and Pearson’s correlations for mental health outcomes (HSCL-37A) and PTE total (SLESQ)*

| Variable | *M* | *SD* | 1 | 2 | 3 | 4 | 5 | 6 | 7 | | 8 |
| --- | --- | --- | --- | --- | --- | --- | --- | --- | --- | --- | --- |
| 1. Intern. Sympoms | 42.30 | 12.98 |  |  |  |  |  |  |  | |  |
| 2. Extern. Symptoms | 16.46 | 5.03 | .74^***^ |  |  |  |  |  |  | |  |
| 3. Depression | 25.95 | 8.41 | .97^***^ | .70^***^ |  |  |  |  |  | |  |
| 4. Anxiety | 16.36 | 5.19 | .93^***^ | .73^***^ | .81^***^ |  |  |  |  | |  |
| 5. Conduct Disorder | 6.36 | 2.08 | .62^***^ | .88^***^ | .58^***^ | .62^***^ |  |  |  | |  |
| 6. Substance Use | 8.72 | 3.46 | .80^***^ | .86^***^ | .78^***^ | .73^***^ | .62^***^ |  |  | |  |
| 7. Oppositional Defiant Disorder | 3.56 | 1.35 | .52^***^ | .65^***^ | .50^***^ | .49^***^ | .48^***^ | .42^***^ |  | |  |
| 8. Post-migration Stress | 19.90 | 5.71 | .42^***^ | .33^***^ | .41^***^ | .40^***^ | .24^**^ | .41^***^ | .21^**^ | |  |
| 9. PTE Total | 4.53 | 2.65 | .36^***^ | .25^**^ | .36^***^ | .31^***^ | .19^*^ | .32^***^ | .20^*^ | .10 | |

*Note.* *M* and *SD* are used to represent mean and standard deviation, respectively.
* *p* < .05. ** *p* < .01. *** *p* < .001. Depression, Anxiety, Intern. Symptoms, Extern. Symptoms = HSCL-37A depression, anxiety, internalizing symptoms, externalizing symptoms subscale outcome; PTE Total = SLESQ Sum score.

Table S3

*Descriptive statistics and correlations for PTEs (SLESQ items), internalizing and externalizing symptoms (HSCL-37A subscale outcome)*

| **Variable** | ***M*** | ***SD*** |  | **1** | **2** | **3** | **4** | **5** | **6** | **7** | **8** | **9** | **10** | **11** | **12** | **13** | **14** | **15** | **16** | **17** | **18** | **19** |
| --- | --- | --- | --- | --- | --- | --- | --- | --- | --- | --- | --- | --- | --- | --- | --- | --- | --- | --- | --- | --- | --- | --- |
| **1. PTE 1** | 0.50 | ^*^0.50 |  |  |  |  |  |  |  |  |  |  |  |  |  |  |  |  |  |  |  |  |
| **2. PTE 2** | 0.19 | ^*^0.39 |  | .24^**^ |  |  |  |  |  |  |  |  |  |  |  |  |  |  |  |  |  |  |
| **3. PTE 3** | 0.51 | ^*^0.50 |  | -.02 | -.01 |  |  |  |  |  |  |  |  |  |  |  |  |  |  |  |  |  |
| **4. PTE 4** | 0.21 | ^*^0.41 |  | ^*^.11 | .10 | -.05 |  |  |  |  |  |  |  |  |  |  |  |  |  |  |  |  |
| **5. PTE 5** | 0.36 | ^*^0.48 |  | ^*^.22^**^ | .28^**^ | ^*^.21^*^ | ^*^.03 |  |  |  |  |  |  |  |  |  |  |  |  |  |  |  |
| **6. PTE 6** | 0.27 | ^*^0.45 |  | ^*^.07 | .21^*^ | ^*^.03 | ^*^.13 | .26^**^ |  |  |  |  |  |  |  |  |  |  |  |  |  |  |
| **7. PTE 7** | 0.67 | ^*^0.47 |  | ^*^.09 | .12 | ^*^.01 | -.02 | .11 | -.08 |  |  |  |  |  |  |  |  |  |  |  |  |  |
| **8. PTE 8** | 0.37 | ^*^0.48 |  | ^*^.19^*^ | .20^*^ | ^*^.15 | ^*^.09 | .26^**^ | ^*^.06 | ^*^.28^**^ |  |  |  |  |  |  |  |  |  |  |  |  |
| **9. PTE 9** | 0.43 | ^*^0.50 |  | ^*^.22^**^ | .14 | ^*^.13 | ^*^.19^*^ | .27^**^ | ^*^.22^**^ | ^*^.02 | .30^**^ |  |  |  |  |  |  |  |  |  |  |  |
| **10. PTE 10** | 0.11 | ^*^0.31 |  | ^*^.17^*^ | .39^**^ | ^*^.04 | ^*^.30^**^ | .15 | ^*^.27^**^ | -.07 | .10 | .16 |  |  |  |  |  |  |  |  |  |  |
| **11. PTE 11** | 0.46 | ^*^0.50 |  | ^*^.13 | .14 | ^*^.27^**^ | ^*^.15 | .33^**^ | ^*^.18^*^ | ^*^.20^*^ | .25^**^ | .29^**^ | .16 |  |  |  |  |  |  |  |  |  |
| **12. PTE 12** | 0.46 | ^*^0.50 |  | ^*^.04 | .18^*^ | ^*^.31^**^ | ^*^.03 | .29^**^ | ^*^.22^**^ | ^*^.14 | .15 | .20^*^ | .12 | .56^**^ |  |  |  |  |  |  |  |  |
| **13. Int. Symptoms** | 42.30 | 12.98 |  | ^*^.23^**^ | .32^**^ | ^*^.09 | ^*^.22^**^ | .19^*^ | ^*^.20^**^ | ^*^.01 | .20^*^ | .14 | .25^**^ | .19^*^ | .11 |  |  |  |  |  |  |  |
| **14. Ext. Symptoms** | 16.46 | ^*^5.03 |  | .15 | .25^**^ | ^*^.05 | .19^*^ | .15 | .16 | -.08 | .18^*^ | .14 | .18^*^ | .11 | .06 | .74^**^ |  |  |  |  |  |  |
| **15. Depression** | 25.95 | ^*^8.41 |  | .22^**^ | .34^**^ | ^*^.09 | .17^*^ | .18^*^ | .20^*^ | ^*^.02 | .19^*^ | .16 | .24^**^ | .20^*^ | .12 | .97^**^ | .70^**^ |  |  |  |  |  |
| **16. Anxiety** | 16.36 | ^*^5.19 |  | .22^**^ | .27^**^ | ^*^.09 | .27^**^ | .18^*^ | .18^*^ | -.02 | .18^*^ | .09 | .23^**^ | .15 | .07 | .93^**^ | .73^**^ | .82^**^ |  |  |  |  |
| **17. Cond. Disorder** | 6.36 | ^*^2.08 |  | ^*^.15 | .21^*^ | -.01 | .18^*^ | .04 | .11 | -.07 | .11 | .09 | ^*^.20^*^ | .06 | .10 | .62^**^ | .88^**^ | .58^**^ | .62^**^ |  |  |  |
| **18. Subst. Use** | 7.12 | ^*^2.59 |  | ^*^.13 | .27^**^ | ^*^.06 | .20^*^ | .20^*^ | .18^*^ | -.02 | .24^**^ | .20^*^ | ^*^.17^*^ | .17^*^ | .11 | .80^**^ | .86^**^ | .78^**^ | .73^**^ | .62^**^ |  |  |
| **19. Opp. Disorder** | 3.56 | ^*^1.35 |  | ^*^.09 | .20^*^ | ^*^.17^*^ | -.01 | .22^**^ | .10 | -.10 | .09 | .05 | ^*^.14 | .16^*^ | .06 | .52^**^ | .65^**^ | .50^**^ | .49^**^ | .48^**^ | .42^**^ |  |
| **20. Post-mig. Stress** | 19.90 | ^*^5.71 |  | ^*^.13 | .17^*^ | -.09 | .06 | .05 | -.02 | ^*^.03 | .19^*^ | .12 | -.01 | .01 | .01 | .42^**^ | .33^**^ | .41^**^ | .40^**^ | .24^**^ | .41^**^ | .21^**^ |

*Note.* *M* and *SD* are used to represent mean and standard deviation, respectively. * indicates *p* < .05. ** indicates *p* < .01.
Int. Symptoms = Internalizing Symptoms, Ext. Symptoms = Externalizing Symptoms, Cond. Disorder = Conduct Disorder, Subst. Use = Substance Use; Opp. Disorder = Oppositional Defiant Disorder; Post-mig. Stress = Post-Migration Stress. Point biserial correlations between SLESQ items (#1 – #12) and HSCL-37A outcomes (#13 – #19); *Spearman’s Rho* (ρ) correlations between SLESQ items.
